# On the Traceability of the Hazelnut Production Chain by Means of Trace Elements

**DOI:** 10.3390/molecules27123854

**Published:** 2022-06-15

**Authors:** Elisa Calà, Andrea Fracchia, Elisa Robotti, Federica Gulino, Francesca Gullo, Matteo Oddone, Marco Massacane, Gianluigi Cordone, Maurizio Aceto

**Affiliations:** 1 Dipartimento per lo Sviluppo Sostenibile e la Transizione Ecologica, Università degli Studi del Piemonte Orientale, 5-13100 Vercelli, Italy; elisa.cala@uniupo.it (E.C.); federica.gulino@uniupo.it (F.G.); francesca.gullo@uniupo.it (F.G.); 2 Dipartimento di Scienze e Innovazione Tecnologica, Università degli Studi del Piemonte Orientale, 11-15121 Alessandria, Italy; 20023846@studenti.uniupo.it (A.F.); elisa.robotti@uniupo.it (E.R.); 3 Thermo Fisher Scientific, 20090 Rodano, Italy; matteo.oddone@thermofisher.com; 4 Elah Dufour Spa, 73-15067 Novi Ligure, Italy; 20006439@studenti.uniupo.it (M.M.); gcordone@elah-dufour.it (G.C.)

**Keywords:** ICP-MS, traceability, hazelnuts, PCA, production chain

## Abstract

The production chain of hazelnuts has been studied by analyzing three sets of samples produced in purity from three different pools of hazelnuts of cultivar “Tonda Gentile Trilobata”, “Tonda Gentile Romana” and “Mortarella”, all cultivated in Italy. From each pool, five processed products were obtained: roasted hazelnuts, hazelnut paste, hazelnut cream, Gianduja paste and Gianduiotto paste. After pre-treatment by means of dry ashing, all samples from each cultivar, including raw hazelnuts, were then analyzed by means of Inductively Coupled Plasma–Optical Emission Spectroscopy (ICP-OES) and Inductively Coupled Plasma–Mass Spectrometry (ICP-MS). A good discrimination was obtained among the different chain stages according to the distribution of the trace elements, as expected. More interesting was the discrimination among the different cultivars: it was possible to distinguish the samples produced from the respective cultivar by means of specific chemical markers, particularly Mo and Ni.

## 1. Introduction

The concept of traceability is currently very important in the food market, and the contribution of analytical chemistry has become relevant thanks to the development of methods that can support the actual traceability systems based as the monitoring of material flows. The possibility of tracing the production chain of foodstuffs on a chemical basis is interesting both for consumers, who can be aware of the provenance of the products they purchase, and for producers, who can guarantee protection from fraud. Traceability can be obtained by analytical methods provided that reliable chemical descriptors are identified, providing an ideal fingerprint that is maintained through all the steps of the entire production chain.

This possibility is, however, limited by the availability of reliable markers that could be used to follow the chain from the initial stages to the final product: this can be easier in cases where raw materials are little or not at all modified, such as for most fruit and vegetable products, as it is shown in a recent study on extra virgin olive oil [1]. It can be indeed more difficult in cases where the production chain involves many steps that deeply modify raw materials with additions of other materials and/or technological modifications: examples on wine [2,3] and milk [4] can illustrate this point. On the biological side, DNA-based tools, recently reviewed by Böhme et al. [5], such as DNA barcoding, can constitute a powerful resource in this field. However, very few studies exist in the field of analytical chemistry dealing with this topic.

With particular regards to the confectionery industry, one of the most relevant foodstuffs is provided by hazelnuts (*Corylus avellana* L.). It is well known that the “Tonda Gentile Trilobata” (TGT) variety cultivated in Piemonte (Italy) is considered perhaps the best in the world as far as sensorial characteristics and technological properties are concerned. Moreover, it is characterized by a higher price if compared to other cultivars and its use is, therefore, prone to commercial fraud. There is, therefore, an interest in verifying its real use in processed products that are mostly products mixed with cocoa (pralines, pastas, bars, etc.) and other confectionery ingredients. The precise identification of the cultivar in raw hazelnuts is consolidated, as it can be seen in recent works [6,7]. The link between soil and fruit, based on the distribution of lanthanides, has been established in previous traceability studies [8]. As far as processed products are involved, however, there are no complete studies based on chemical descriptors. Locatelli et al. [9] studied the effect of roasting on the possibility of recognizing different cultivars, particularly TGT, in hazelnuts isolated from commercial products. Torello Marinoni et al. [10] were able to follow the production chain of hazelnut from the fruit up to processed products, exploiting specific types of biological descriptors such as nuclear SSR (simple sequence repeats), SNP (single nucleotide polymorphism), InDels (insertions and deletions) and chloroplast markers. The request of recognizing the use of TGT in ready-to-eat confectionery products using methods based on analytical chemistry is at present unaddressed.

In this work, the production chain of hazelnuts has been studied by analyzing three sets of samples produced in purity from three different pools of hazelnuts. The hazelnuts pools were of cultivar “Tonda Gentile Trilobata” (TGT), “Tonda Gentile Romana” (TGR) and “Mortarella” (MOR). From each pool, five processed products were obtained: roasted hazelnuts, hazelnut paste, hazelnut cream (a spreadable cream made from a mixture of hazelnut paste and cocoa), Gianduja paste (the same as hazelnut cream but richer in cocoa) and Gianduiotto paste (similarly to Gianduja paste but used to prepare a typical Piemontese chocolate candy called Gianduiotto). All five products from each cultivar and raw hazelnuts, as well a total of 18 samples, were then analyzed by means of Inductively Coupled Plasma–Optical Emission Spectroscopy (ICP-OES) and Inductively Coupled Plasma–Mass Spectrometry (ICP-MS), attempting to verify whether the determined distribution of the trace elements was able to recognize the original cultivars used in the processed products.

## 2. Results and Discussion

**Figure 1:**
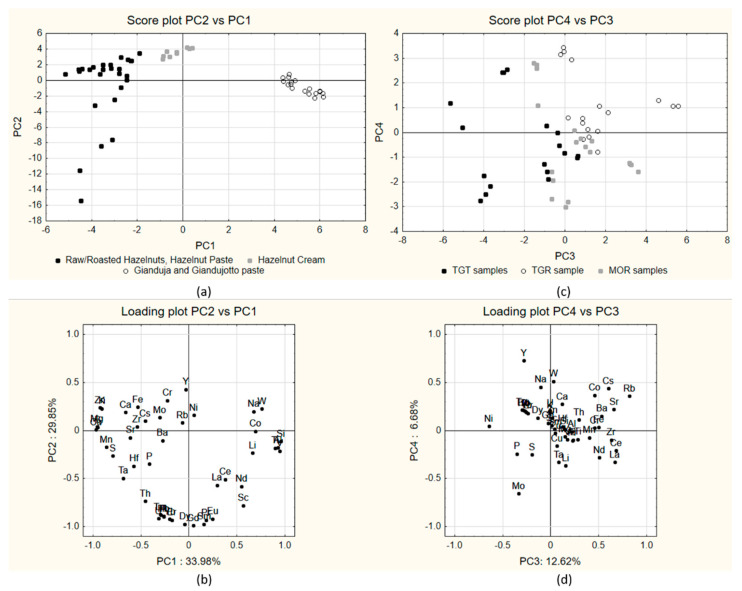
Results of PCA of the 54 samples, based on all variables: score plot (**a**) and loading plot (**b**) of PC1 vs. PC2; score plot (**c**) and loading plot (**d**) of PC3 vs. PC4.

The 54 samples (3 cultivar × 6 products × 3 independent replicates) were analyzed by means of ICP-OES and ICP-MS analysis and yielded the data reported in Table S1. All 46 elements determined were above the LOQ (Limit of Quantification) values for the instruments used in this work. Elemental data were subjected to multivariate analysis in order to evaluate potential groupings; the dataset comrpised 54 samples × 46 variables, which were transformed into z-scores before analysis. A reduction in dimensionality by means of PCA yielded the results shown in Figure 1. The first two PCs accounted for the 63.83% of the total explained variance and showed no clusterization of the samples according to cultivar but rather according to the product type (Figure 1a): the samples can be divided into three main groups:

1. Cluster 1, including products containing only hazelnuts (raw, roasted and paste, hereafter termed *hazelnuts-only products*), clustering at negative values of PC1 and negative and positive values on PC2 (black dots in figure);
2. Cluster 2, including hazelnut cream samples, characterized by the presence of small amounts of cocoa powder, clustered at intermediate values on PC1 and positive scores on PC2;
3. Cluster 3, including Gianduja and Gianduiotto paste samples (white dots in figure), characterized by higher amounts of cocoa powder and clustering mostly at positive values of PC1 and negative or slightly negative values of PC2.

The observed clustering can be attributed to the increasing contribution of cocoa, absent in hazelnuts-only products, but added to hazelnut cream, Gianduja and Gianduiotto paste (the last two in larger percentages), as it emerges from the content of the two main ingredients (hazelnuts and cocoa) of the different products (Table 1).

In fact, as it emerges from the loadings (Figure 1b), the elements positively contributing to this difference are Al, Co, Li, Na and Si, which, according to the literature data (Table 2), are higher in cocoa than in hazelnuts. In particular, silicon in cocoa beans is estimated to be up to 300 times higher than in hazelnuts [11]; as for aluminum, in the study by Stahl et al. [12], the authors found that cocoa powder was among the foodstuffs with the higher Al content, which is possibly due to the contribution of growing soil.

To resume, it seems that a good correlation exists between the mineral content of our samples and their cocoa powder %.

Regarding to the information about the separation of the three cultivars, it seems mostly related to PC3 and PC4 (Figure 1c), accounting for about 20% of the variance: The samples appear, in fact, quite clustered according to the cultivar with TGT samples at negative scores on PC3, TGR samples at positive scores on both PC3 and PC4 and MOR samples at intermediate cores on PC3. Looking at the corresponding loading plot (Figure 1d), Ni, Mo, Cs and Rb appeared as the variables mostly related to this separation (Ni and Mo more present in TGT samples, while Cs and Rb more present in TGR samples).

### 2.1. Grouping by Process Stage

**Figure 2:**
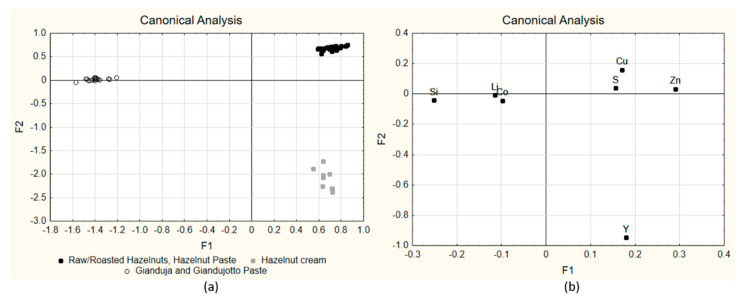
LDA score plot of the 54 samples, F1 vs. F2 for the classification according to the product type: score plot of the canonical variables (**a**); loading plot of the canonical variables (**b**).

The effect of processing was then investigated by looking for chemical markers able to discriminate among the different processed products. Applying LDA to the elemental data with variable selection in forward search (T_to-enter_ = 3, F_to-remove_ = 2), seven variables were selected as significant (α < 0.05). A good classification was obtained, as expected, and all samples were correctly classified both in fitting and in cross-validation (Monte Carlo with elimination of the 20% of the objects of each class at each iteration; 100 iterations); the elements selected by the forward search procedure were Y, Zn, Cu, Si, Li, S and Co. The calculated canonical functions allowed the obtainment of the F1 vs. F2 diagrams reported in Figure 2a, where hazelnuts-only products (Cluster 1) obtained positive scores on both F1 and F2, Hazelnut cream (Cluster 2) obtained positive scores on F1 and negative ones on F2 and Gianduja and Gianduiotto pastes (Cluster 3) obtained negative scores on F1. Looking at the corresponding loadings (Figure 2b), Group 1 and 2 are characterized by high values of Cu, S Zn and Y and low levels of Li, Si and Co if compared to Cluster 3, but Cluster 2 shows a larger concentration of Y and lower concentration of Cu, S and Zn, while Cluster 1 has an opposite behavior. Cluster 3 shows instead a high content of Li, Si and Co and low content of the other elements (Cu, S, Zn and Y). As explained before, the presence of some of these descriptors in the discrimination model is due to their different contents in hazelnut or cocoa powder.

### 2.2. Grouping by Cultivar

After discriminating according to the type of product/production chain stage, the possibility of discriminating products was verified according to the original hazelnut cultivars, i.e., “Tonda Gentile Trilobata” (TGT), “Tonda Gentile Romana” (TGR) and “Mortarella” (MOR). This is the most important and useful discrimination, as it would allow the verification of the origin of the raw materials even at the end of the production chain. In particular, verifying the possibility of discriminating between products obtained from TGT hazelnuts and products obtained from other hazelnuts was important.

**Figure 3:**
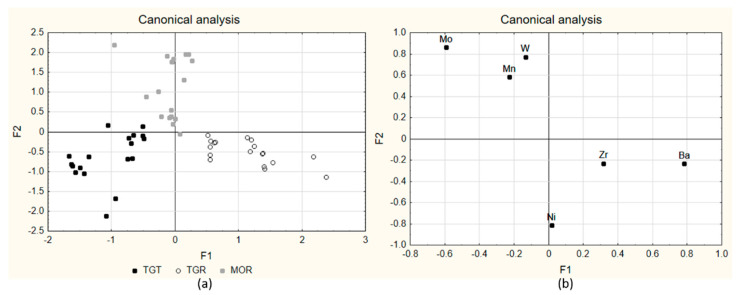
LDA score plot of the 54 samples, F1 vs. F2 for the classification according to the cultivar: score plot of the canonical variables (**a**); loading plot of the canonical variables (**b**).

The preliminary results of the reduction in dimensionality by means of PCA, shown in Figure 1, were unsatisfactory from the point of view of the discrimination conducted among cultivars, because, as explained before, the samples were clustered, independently from the cultivar, into three groups: (1) hazelnut-only products; (2) hazelnut cream samples; (3) Gianduja pastes/Gianduiotto pastes. Therefore, LDA was again used in order to achieve a better classification, with variable selection in forward search (T_to-enter_ = 3, F_to-remove_ = 2). Six variables were selected as significant (α < 0.05). The calculated canonical functions allowed obtaining F1 vs. F2 diagrams reported in Figure 3a, which show very good clustering in which all samples were grouped according to the original cultivar.

All 54 samples were correctly classified in fitting and very good performances were reached in cross-validation (Monte Carlo with elimination of the 20% of the objects of each class at each iteration; 100 iterations) with NER% = 94.82%. The performance indexes are reported in Table 3.

The selected variables were Mn, Ni, Zr, Mo, Ba and W.

**Figure 4:**
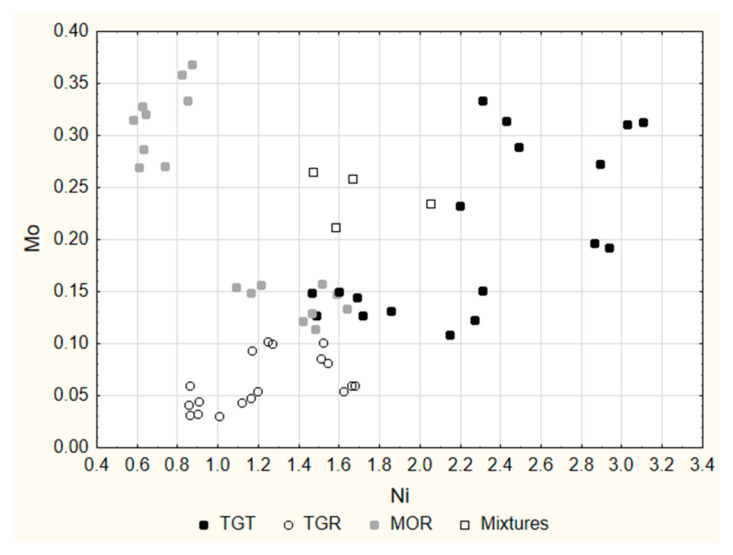
Two-dimensional plot of the 54 samples: Mo vs. Ni.

This behavior seems to partly reflect the elemental content of the hazelnut component. In fact, the application of LDA to a dataset containing the hazelnut-only products (data in Figure S1) highlighted that Mo, Ni and Sr were the more efficient elements in the discrimination among the three cultivars, with a minor role of Ce, La, Mn and Rb. We may hypothesize that the addition of cocoa powder and other ingredients does not influence the original content of these elements, while it somewhat influences the original content of Ba and Cs. Following the indications of LDA, Mo and Ni were selected among the variables with the highest discriminating power and their 2D plot (Figure 4) allowed obtaining a good separation among the three cultivars.

In the end, therefore, Mo, and Ni worked efficiently as the chemical markers of the original hazelnut cultivars.

Similar results were achieved after removing the information about the type of product, operating a centering of the data according to the three types of products classes separately.

### 2.3. Verivication with Samples Prepared in Laboratory

A first attempt to evaluate the suitability of the method was made by preparing two samples of Gianduja pastes and two samples of Gianduiotto pastes with variable amounts of hazelnuts of known origin (Table 4). All four paste samples have features somewhat intermediate among the three cultivars, with samples Gianduja 1 and Gianduiotto 1 possessing a higher percentage of Piemonte hazelnuts.

The four samples were subjected to the same treatment and analyzed in the same conditions by means of ICP-MS and ICP-OES. The results were then compared with previous ones (see Figure 4), and they confirmed that Mo and Ni are good chemical markers for distinguishing hazelnut cultivars.

## 3. Materials and Methods

### 3.1. Materials

High-purity water (HPW) with resistance > 18 MΩ·cm was produced with a Milli-Q (Milford, MA, USA) apparatus. Ultrapure nitric and hydrochloric acids and 30% hydrogen peroxide, all of TraceSelect grade, were purchased from Fluka (Milan, Italy). Multielement standard solutions for ICP analysis were purchased from Inorganic Ventures (Christiansburg, VA, USA).

### 3.2. Sample Collection

Samples of raw hazelnuts, roasted hazelnuts, hazelnut paste, hazelnut cream, Gianduja paste and Gianduiotto paste for each of the three cultivar “Tonda Gentile Trilobata” (TGT), “Tonda Gentile Romana” (TGR) and “Mortarella” (MOR) were provided by Elah Dufour Novi (Novi Ligure, Italy). The samples had the following features:

1. Raw hazelnuts: 1 Kg of hazelnuts was collected and placed under vacuum;
2. Roasted hazelnuts: after roasting, 1 Kg of hazelnuts was collected and placed under vacuum;
3. Hazelnut paste: Roasted hazelnuts were mashed in order to obtain a paste that subsequently divided into a supernatant fraction rich in lipids and with lower solid fraction; both were kept in a polyethylene bottle;
4. Hazelnut cream: After processing the hazelnut paste, 0.5 Kg of cream was taken and kept in a polyethylene bottle;
5. Gianduja paste: After processing the hazelnut paste, 0.5 Kg of product was taken and kept in a polyethylene bottle;
6. Gianduiotto paste: After processing the hazelnut paste, 0.5 Kg of product was taken and kept in a polyethylene bottle.

For every product, three independent replicates were collected and treated for a total of 54 samples.

### 3.3. Sample Treatment

All samples were subjected to dry ashing supported by microwave irradiation by using a Milestone (Sorisole, Italy) Pyro 260 microwave ashing system. Dry ashing was chosen as pre-treatment instead of acid digestion, because it allows treating a higher amount of sample (usually 10× with respect to acid digestion), maximizing the content of trace elements in the solutions to be analyzed. Its major drawback is the loss of volatile elements (e.g., As, Cd, Hg and Pb) but we evaluated that they were not relevant descriptors for traceability.

For dry ashing, 20 g of each sample was weighed and placed in a porcelain capsule. The heating cycle was as follows: room temperature to 150 °C in 10′; hold at 150 °C for 20′; up to 500 °C in 20′; hold at 500 °C for 30′; up to 750 °C in 10′; hold at 750 °C for 30′; up to 900 °C (raw hazelnuts, roasted hazelnuts, hazelnut paste and hazelnut cream) or to 1000 °C (Gianduja and Gianduiotto paste) in 10′; hold at 900 °C (raw hazelnuts, roasted hazelnuts, hazelnut paste and hazelnut cream) or to 1000 °C (Gianduja and Gianduiotto paste) for 30′. The higher final temperature for Gianduja and Gianduiotto paste was needed in order to obtain a more efficient incineration. The resulting ash was completely dissolved in 2.0 mL of ultrapure concentrated nitric acid and taken up to 45 mL with HPW in a polypropylene tube.

### 3.4. ICP-OES Analysis

The determination of major and minor elements was carried out with a Spectro (SPECTRO Analytical Instruments GmbH, Kleve, Germany) Genesis ICP-OES simultaneous spectrometer with axial plasma observation. Instrumental parameters were as follows: pump speed, 2.0 mL/min; RF generator, 40 MHz; RF, 1300 W; plasma power, 1400 W; plasma gas outlet, 12 L/min; auxiliary gas flow rate, 0.90 L/min; nebulizer flow rate, 0.96 L/min. The elements determined were the following (in parentheses the ƒ⊄ of acquisition): Al (396.152 nm), B (249.773 nm), Ca (317.993 nm), Co (228.616 nm), Cu (324.754 nm), Fe (259.941 nm), K (766.491 nm), Li (670.780 nm), Mg (285.213 nm), Mn (257.611 nm), Na (589.592 nm), Ni (231.604 nm), P (177.495 nm), Rb (420.185 nm), S (180.731 nm), Si (251.612 nm), Sr (460.733 nm) and Zn (213.856 nm). CCS-4 and CCS-5 multi-element standard solutions from Inorganic Ventures (Christiansburg, VA, USA) were used to prepare 10, 5, 1, 0.5 and 0.1 mg/L solutions in 1% nitric acid. Limits of detection (LOD) and limits of quantification (LOQ), calculated as, respectively, 3 and 10 times the standard deviation of blank measurements, can be found in Table S2.

### 3.5. ICP-MS Analysis

The determination of trace and ultra-trace elements was carried out with a Thermo Scientific (Waltham, MA, USA) iCAP^TM^ RQ inductively coupled plasma mass spectrometer with single quadrupole technology. The instrument is equipped with an ESI (Omaha, NE, USA) PFA 100 MicroFlow nebulizer, a Peltier-cooled quartz spray chamber operating at 3 °C, a 2.0 mm ID quartz injector and a demountable quartz torch. Measurements were carried out by exploiting an ESI (Omaha, NE, USA) SC-4 DX autosampler. To overcome spectral interferences, the Collision Cell Technology (CCT) was used with He gas at 3.5 mL/min and a kinetic energy discrimination (KED) barrier of 2 V; the CCT-KED device was particularly useful in minimizing the interferences of oxides (e.g., ^141^Pr^16^O on ^157^Gd) in the determination of lanthanides. Sensitivity performances were comparable between standard and KED mode (Ce > 500k cps/ppb in both modes) thanks to the extraordinary efficiency of Qcell flatpole; therefore, only the KED experimental setting was used. Instrument and accessories were PC-controlled by Qtegra^TM^ v. 2.10.4345.136 software. Instrumental parameters were as follows: forward power, 1550 W; plasma gas flow, 14.0 L/min; nebulizer gas flow, 0.9 L/min; auxiliary gas flow, 0.8 L/min. Three replicates were made, for a total acquisition time of 180 s. The following isotopes were used: ^45^Sc, ^47^Ti, ^51^V, ^52^Cr, ^89^Y, ^90^Zr, ^95^Mo, ^133^Cs, ^137^Ba, ^139^La, ^140^Ce, ^141^Pr, ^146^Nd, ^147^Sm, ^151^Eu, ^157^Gd, ^159^Tb, ^163^Dy, ^165^Ho, ^166^Er, ^169^Tm, ^172^Yb, ^175^Lu, ^178^Hf, ^181^Ta, ^182^W, ^232^Th and ^238^U. ^103^Rh, ^115^In and ^193^Ir were used as internal standards.

Interference due to oxide formation was evaluated as follows: CeO^+^/Ce^+^ < 0.5% in KED mode. A stability test performed before each session by monitoring ^7^Li, ^59^Co, ^115^In, ^140^Ce and ^238^U, which yielded a precision higher than 2%. The instrumental precision was better than 2% for trace and ultra-trace elements, while the overall precision, involving both sample preparation and instrumental analysis, was better than 5%, as calculated on five genuine replicates. Background signals were monitored at 5, 101 and 220 m/z to perform a sensitivity test on the above-reported analyte masses. CCS-1, CCS-2, CCS-4, CCS-5 and CCS-6 multi-element standard solutions from Inorganic Ventures (Christiansburg, VA, USA) were used to prepare 100, 10, 1 and 0.1 µg/L solutions in 1% nitric acid. Internal standardization monitoring relative to ^103^Rh, ^115^In and ^193^Ir isotopes was used to correct for instrumental drifts by means of interpolation to yield a better correction; the three isotopes were added to all solutions analyzed at 10 μg/L. The limits of detection (LOD) and limits of quantification (LOQ), calculated as respectively 3 and 10 times the standard deviation of blank measurements, can be found in Table S2.

### 3.6. Analysis of Certified Samples

No certified materials exist for hazelnut nor for its processed products.

### 3.7. Data Analysis

PCA and LDA models were carried out by Statistica v.7.1 (Statsoft, Tulsa, OK, USA) and Matlab R2014a (The Mathworks, Natick, MS, USA); graphical representations were carried out by Statistica v.7.1 (Statsoft, Tulsa, OK, USA). Data were autoscaled before PCA. LDA was carried out exploiting variable selection by a forward search algorithm (F_to-nter_ = 3, F_to-remove_ = 1). Cross-validation was applied by the Monte Carlo method with 20% of object of each class eliminated at each iteration for 100 iterations.

## 4. Conclusions

The elemental analysis of confectionery products along the production chain of hazelnuts and cocoa based products was shown to be useful in their characterization and proved to be affective in establishing their traceability relative to the cultivar of the starting raw hazelnuts. It was possible to discriminate among the various stages of the production chain according to the content of Y, Zn, Cu, Si, Li, S and Co and, what it is more interesting, among the different cultivars used as raw materials according to the content of Mo, Ni, Ba, Mn, Zr and W. Further experiments are needed in order to address to different points: (1) verify the possibility of discrimination among the cultivars including other cultivars and increasing the number of samples for each cultivar; (2) explore more exhaustively the possibility to discriminate the cultivars also in presence of mixtures of different cultivars.

## Figures and Tables

**Table 1:** Average compositions of the products.

Product	Hazelnut %	Cocoa %
raw hazelnuts	100	0
roasted hazelnuts	100	0
hazelnut paste	100	0
hazelnut cream	45	12
Gianduja paste	20	34
Gianduiotto paste	26	24

**Table 2:** Mineral content of hazelnuts and cocoa powder from the literature data.

Element	Hazelnut (mg/Kg)	Cocoa (mg/Kg)
Al	0.9–18 [13]	41–275 [14]
Co	0.07–0.6 [13]	0.4–0.6 [14]
Li	0.035–0.042 [15]	0.01–0.05 [14]
Na	0.06–5 [6]	10–32 [14]
Si	13 [16]	400–4700 [11]
Ba	2–23 [6]	5.9–22.2 [14]
Mo	0.09–0.31 [13]	0.1–0.4 [14]
Ni	0.58–2.58 [15]	4.9–12.1 [14]
Sr	4–23 [6]	6.8–18.1 [14]

**Table 3:** Precision, Sensitivity and Specificity achieved in cross-validation by the final model containing 6 variables.

Samples	Precision	Sensitivity	Specificity
TGT	94.92%	93.33%	97.57%
TGR	95.69%	95.97%	98.01%
MOR	93.97%	95.17%	96.61%

**Table 4:** Samples of Gianduja and Gianduiotto pastes prepared with mixtures of hazelnuts of known origin. The column “Hazelnuts %” indicates the total amount of hazelnuts in the samples.

Sample	Piemonte %	Mortarella %	Romana %	Hazelnut %
Gianduja mix 1	50	25	25	21
Gianduja mix 2	33	33	33	27
Gianduiotto mix 1	50	25	25	27
Gianduiotto mix 2	33	33	33	35

## Data Availability

Not applicable.

## Supplementary Materials

The following supporting information can be downloaded at: https://www.mdpi.com/article/10.3390/molecules27123854/s1, Figure S1: LDA score plot of the 27 samples of hazelnut-only products, F1 vs. F2 for the classification according to the cultivar: score plot of the canonical variables; Table S1: Concentrations of analytes (mg/Kg) determined by means of ICP-OES and ICP-MS; Table S2: LOD and LOQ for the elements determined with ICP-OES and ICP-MS.
