# Supplementary material for: On the Traceability of the Hazelnut Production Chain by Means of Trace Elements
Source: Molecules. 2022 Jun 15;27(12):3854. doi: 10.3390/molecules27123854 (PMC9228825; doi:10.3390/molecules27123854)
Supplement: Supplementary file 1 [file molecules-27-03854-s001.zip › Table S1.pdf]

Samples	Al	B	Ba	Ca	Ce	Co	Cr	Cs	Cu
Piemonte raw hazelnuts	3.57	14.55	0.87	1351.1	0.0059	0.2201	0.0128	0.00002	16.34
Romana raw hazelnuts	5.33	16.53	2.35	1475.0	0.0146	0.2809	0.0071	0.00019	17.24
Mortarella raw hazelnuts	5.06	14.92	2.82	1202.0	0.0116	0.1955	0.0070	0.00007	17.37
Piemonte roasted hazelnuts	1.98	12.91	0.70	1278.8	0.0048	0.2109	0.0072	0.00003	15.92
Romana roasted hazelnuts	2.87	16.27	3.83	1381.8	0.0080	0.2775	0.0053	0.00034	15.74
Mortarella roasted hazelnuts	3.31	15.84	3.04	1374.3	0.0089	0.2106	0.0060	0.00008	17.91
Piemonte hazelnut paste	2.47	18.67	0.90	1587.5	0.0079	0.2995	0.2525	0.00003	18.47
Romana hazelnut paste	3.89	22.09	2.23	1632.4	0.0110	0.3755	0.2301	0.00042	19.44
Mortarella hazelnut paste	3.47	19.73	3.20	1548.8	0.0108	0.2740	0.2632	0.00010	17.66
Piemonte hazelnut cream	4.90	9.66	0.78	1385.3	0.0053	0.3007	0.0810	0.00006	11.21
Romana hazelnut cream	5.57	11.81	3.18	1443.8	0.0078	0.3495	0.0846	0.00025	13.38
Mortarella hazelnut cream	5.60	10.87	1.75	1441.1	0.0067	0.3100	0.0825	0.00011	12.78
Piemonte Gianduja paste	12.71	5.30	1.16	873.6	0.0131	0.3877	0.0555	0.00002	7.56
Romana Gianduja paste	13.49	5.66	2.27	891.2	0.0135	0.4061	0.0498	0.00007	7.67
Mortarella Gianduja paste	13.15	5.63	1.84	885.4	0.0130	0.3988	0.0590	0.00005	7.99
Piemonte Gianduiotto paste	9.38	5.40	0.91	1388.5	0.0099	0.3484	0.0571	0.00002	7.30
Romana Gianduiotto paste	9.48	6.14	2.37	1395.0	0.0110	0.3711	0.0577	0.00008	7.76
Mortarella Gianduiotto paste	9.75	6.12	1.87	1424.0	0.0117	0.3564	0.0689	0.00006	8.15

Table S1a. Concentrations of analytes (mg/Kg) determined by means of ICP-OES and ICP-MS.

Samples	Dy	Er	Eu	Fe	Gd	Hf	Ho	K	La
Piemonte raw hazelnuts	0.00089	0.00077	0.00120	31.47	0.00098	0.0028	0.00068	2904.9	0.0049
Romana raw hazelnuts	0.00154	0.00125	0.00183	40.10	0.00178	0.0030	0.00112	3036.4	0.0096
Mortarella raw hazelnuts	0.00057	0.00032	0.00086	37.51	0.00076	0.0028	0.00015	3078.3	0.0082
Piemonte roasted hazelnuts	0.00086	0.00076	0.00119	25.48	0.00095	0.0029	0.00072	2853.7	0.0040
Romana roasted hazelnuts	0.00034	0.00017	0.00078	36.89	0.00053	0.0027	0.00008	2892.6	0.0056
Mortarella roasted hazelnuts	0.00109	0.00084	0.00155	40.55	0.00127	0.0029	0.00075	3111.4	0.0065
Piemonte hazelnut paste	0.00034	0.00015	0.00095	85.13	0.00054	0.0027	0.00008	3214.3	0.0060
Romana hazelnut paste	0.00042	0.00020	0.00086	91.89	0.00066	0.0029	0.00010	3358.5	0.0081
Mortarella hazelnut paste	0.00045	0.00021	0.00118	97.99	0.00070	0.0028	0.00010	3366.0	0.0085
Piemonte hazelnut cream	0.00026	0.00014	0.00040	41.79	0.00034	0.0027	0.00007	3108.2	0.0029
Romana hazelnut cream	0.00033	0.00018	0.00065	49.50	0.00046	0.0028	0.00008	3219.9	0.0047
Mortarella hazelnut cream	0.00031	0.00017	0.00051	51.90	0.00041	0.0028	0.00008	3278.3	0.0040
Piemonte Gianduja paste	0.00082	0.00039	0.00187	30.31	0.00114	0.0027	0.00017	2005.2	0.0090
Romana Gianduja paste	0.00082	0.00040	0.00185	34.39	0.00116	0.0027	0.00016	2044.0	0.0090
Mortarella Gianduja paste	0.00079	0.00039	0.00168	33.82	0.00111	0.0027	0.00016	2057.3	0.0089
Piemonte Gianduiotto paste	0.00056	0.00028	0.00126	26.72	0.00081	0.0027	0.00012	2063.0	0.0072
Romana Gianduiotto paste	0.00062	0.00030	0.00118	29.05	0.00085	0.0027	0.00013	2067.8	0.0075
Mortarella Gianduiotto paste	0.00064	0.00032	0.00138	30.35	0.00094	0.0027	0.00013	2110.5	0.0085

Table S1b. Concentrations of analytes (mg/Kg) determined by means of ICP-OES and ICP-MS.

Samples	Li	Lu	Mg	Mn	Mo	Na	Nd	Ni	P	Pr
Piemonte raw hazelnuts	7.01	0.00077	1222.7	31.95	0.2545	104.0	0.0035	2.46	359.5	0.00138
Romana raw hazelnuts	8.23	0.00119	1302.6	56.61	0.0349	132.9	0.0066	0.92	200.1	0.00250
Mortarella raw hazelnuts	7.75	0.00010	1202.4	52.10	0.3044	108.0	0.0051	0.60	110.0	0.00149
Piemonte roasted hazelnuts	7.24	0.00071	1161.4	24.34	0.2649	91.0	0.0029	2.62	74.0	0.00128
Romana roasted hazelnuts	5.35	0.00005	1230.0	49.11	0.0452	77.8	0.0034	0.87	58.5	0.00099
Mortarella roasted hazelnuts	6.35	0.00070	1237.5	50.78	0.2928	85.3	0.0043	0.67	47.4	0.00183
Piemonte hazelnut paste	7.84	0.00005	1359.9	32.78	0.2987	91.0	0.0043	3.01	46.7	0.00122
Romana hazelnut paste	6.00	0.00005	1438.1	59.50	0.0482	78.0	0.0051	1.16	43.9	0.00148
Mortarella hazelnut paste	7.28	0.00005	1381.7	55.64	0.3540	93.9	0.0054	0.85	41.1	0.00156
Piemonte hazelnut cream	5.48	0.00005	1029.7	17.66	0.1274	292.3	0.0021	2.24	32.1	0.00059
Romana hazelnut cream	4.43	0.00005	1121.7	30.49	0.0580	276.2	0.0031	1.65	34.0	0.00088
Mortarella hazelnut cream	5.82	0.00004	1102.4	28.20	0.1463	321.6	0.0027	1.58	34.1	0.00075
Piemonte Gianduja paste	9.44	0.00008	828.5	12.04	0.1288	181.2	0.0076	1.69	20.8	0.00205
Romana Gianduja paste	9.20	0.00008	852.5	15.77	0.0896	180.5	0.0076	1.52	22.0	0.00200
Mortarella Gianduja paste	9.16	0.00008	851.1	15.43	0.1218	195.4	0.0074	1.45	22.7	0.00196
Piemonte Gianduiotto paste	10.98	0.00006	780.9	10.35	0.1480	340.5	0.0058	1.58	28.1	0.00155

Romana Gianduiotto paste	12.17	0.00006	805.3	16.74	0.0987	354.8	0.0058	1.23	29.0	0.00158
Mortarella Gianduiotto paste	12.54	0.00007	808.8	16.38	0.1535	369.5	0.0066	1.15	28.7	0.00179

Table S1c. Concentrations of analytes (mg/Kg) determined by means of ICP-OES and ICP-MS.

Samples	Rb	S	Sc	Si	Sm	Sr	Ta	Tb	Th
Piemonte raw hazelnuts	0.80	198.9	0.0032	2.93	0.0011	7.56	0.00047	0.00066	0.00178
Romana raw hazelnuts	1.71	178.6	0.0044	2.97	0.0020	33.20	0.00050	0.00115	0.00546
Mortarella raw hazelnuts	0.88	142.8	0.0012	3.24	0.0009	18.59	0.00050	0.00016	0.00191
Piemonte roasted hazelnuts	0.93	107.9	0.0015	1.26	0.0011	5.71	0.00047	0.00072	0.00072
Romana roasted hazelnuts	1.85	122.4	0.0010	1.57	0.0006	41.04	0.00047	0.00009	0.00225
Mortarella roasted hazelnuts	1.10	101.7	0.0030	1.92	0.0015	19.27	0.00048	0.00082	0.00214
Piemonte hazelnut paste	1.25	87.2	0.0014	1.95	0.0008	8.20	0.00046	0.00010	0.00066
Romana hazelnut paste	2.22	92.1	0.0014	2.35	0.0009	44.21	0.00046	0.00011	0.00266
Mortarella hazelnut paste	2.13	77.8	0.0011	2.09	0.0010	23.56	0.00047	0.00011	0.00130
Piemonte hazelnut cream	1.34	55.2	0.0014	4.67	0.0004	5.72	0.00041	0.00008	0.00026
Romana hazelnut cream	1.43	56.3	0.0014	5.76	0.0006	21.63	0.00044	0.00008	0.00092
Mortarella hazelnut cream	1.45	50.9	0.0012	5.20	0.0005	12.79	0.00043	0.00008	0.00044
Piemonte Gianduja paste	1.28	26.0	0.0052	14.39	0.0015	6.14	0.00043	0.00018	0.00069
Romana Gianduja paste	1.36	24.7	0.0050	15.41	0.0015	10.19	0.00043	0.00018	0.00110
Mortarella Gianduja paste	1.41	22.1	0.0049	13.63	0.0015	7.78	0.00043	0.00018	0.00088
Piemonte Gianduiotto paste	1.59	20.6	0.0038	11.81	0.0011	4.02	0.00043	0.00013	0.00056
Romana Gianduiotto paste	1.60	20.8	0.0039	12.62	0.0012	11.57	0.00043	0.00014	0.00128
Mortarella Gianduiotto paste	1.64	19.2	0.0041	12.58	0.0013	8.67	0.00043	0.00015	0.00102

Table S1d. Concentrations of analytes (mg/Kg) determined by means of ICP-OES and ICP-MS.

Samples	Ti	Tm	U	V	W	Y	Yb	Zn	Zr
Piemonte raw hazelnuts	0.3482	0.00071	0.0008	0.0058	0.0020	0.0023	0.00080	13.21	0.0046
Romana raw hazelnuts	0.4308	0.00115	0.0017	0.0064	0.0054	0.0056	0.00126	14.98	0.0106
Mortarella raw hazelnuts	0.3934	0.00009	0.0006	0.0047	0.0046	0.0043	0.00027	15.24	0.0054
Piemonte roasted hazelnuts	0.2497	0.00068	0.0006	0.0035	0.0022	0.0018	0.00073	13.76	0.0046
Romana roasted hazelnuts	0.3074	0.00004	0.0006	0.0035	0.0059	0.0029	0.00013	14.78	0.0051
Mortarella roasted hazelnuts	0.3076	0.00069	0.0010	0.0031	0.0046	0.0041	0.00079	15.49	0.0074
Piemonte hazelnut paste	0.3217	0.00004	0.0002	0.0042	0.0076	0.0018	0.00011	19.59	0.0055
Romana hazelnut paste	0.3812	0.00005	0.0006	0.0044	0.0093	0.0037	0.00015	20.84	0.0195
Mortarella hazelnut paste	0.3467	0.00004	0.0004	0.0039	0.0091	0.0036	0.00017	20.42	0.0153
Piemonte hazelnut cream	0.3728	0.00004	0.0002	0.0054	0.0346	0.3023	0.00012	15.41	0.0040
Romana hazelnut cream	0.3811	0.00004	0.0004	0.0060	0.0373	0.2887	0.00014	16.84	0.0036
Mortarella hazelnut cream	0.3740	0.00004	0.0002	0.0053	0.0367	0.3396	0.00013	16.91	0.0048
Piemonte Gianduja paste	0.7410	0.00007	0.0004	0.0219	0.0334	0.0045	0.00032	1.93	0.0036
Romana Gianduja paste	0.7218	0.00007	0.0005	0.0220	0.0327	0.0048	0.00033	1.63	0.0036
Mortarella Gianduja paste	0.7156	0.00007	0.0004	0.0218	0.0338	0.0046	0.00032	1.69	0.0037
Piemonte Gianduiotto paste	0.5800	0.00005	0.0003	0.0161	0.0261	0.0032	0.00022	1.35	0.0038
Romana Gianduiotto paste	0.5888	0.00006	0.0004	0.0160	0.0265	0.0037	0.00025	1.67	0.0036
Mortarella Gianduiotto paste	0.6364	0.00006	0.0004	0.0171	0.0275	0.0040	0.00027	1.28	0.0040

Table S1e. Concentrations of analytes (mg/Kg) determined by means of ICP-OES and ICP-MS.
