# Supplementary material for: On the Traceability of the Hazelnut Production Chain by Means of Trace Elements
Source: Molecules. 2022 Jun 15;27(12):3854. doi: 10.3390/molecules27123854 (PMC9228825; doi:10.3390/molecules27123854)
Supplement: Supplementary file 1 [file molecules-27-03854-s001.zip › Table S2.pdf]

| Element | LOD | LOQ | Element | LOD | LOQ |
|---|---|---|---|---|---|
| Al[1] | 0.027 mg/L | 0.0945 mg/L | Na[1] | 9.9 µg/L | 34.65 µg/L |
| B[1] | 0.004 mg/L | 0.014 mg/L | Nd[2] | 0.2 ng/L | 0.7 ng/L |
| Ba[2] | 0.5 ng/L | 1.75 ng/L | Ni[1] | 1.4 µg/L | 4.9 µg/L |
| Ca[1] | 0.019 mg/L | 0.0665 mg/L | P[1] | 0.24 mg/L | 0.84 mg/L |
| Ce[2] | 0.6 ng/L | 2.1 ng/L | Pr[2] | 0.1 ng/L | 0.35 ng/L |
| Co[1] | 0.67 µg/L | 2.345 µg/L | Rb[1] | 0.035 mg/L | 0.1225 mg/L |
| Cr[2] | 0.001 µg/L | 0.0035 µg/L | S[1] | 0.022 mg/L | 0.077 mg/L |
| Cs[2] | 0.8 ng/L | 2.8 ng/L | Sc[2] | 4.65 ng/L | 16.275 ng/L |
| Cu[1] | 2.5 µg/L | 8.75 µg/L | Si[1] | 4.3 µg/L | 15.05 µg/L |
| Dy[2] | 0.2 ng/L | 0.7 ng/L | Sm[2] | 0.07 ng/L | 0.245 ng/L |
| Er[2] | 0.03 ng/L | 0.105 ng/L | Sr[1] | 0.011 mg/L | 0.0385 mg/L |
| Eu[2] | 0.03 ng/L | 0.105 ng/L | Ta[2] | 1.6 ng/L | 5.6 ng/L |
| Fe[1] | 1.6 µg/L | 5.6 µg/L | Tb[2] | 0.1 ng/L | 0.35 ng/L |
| Gd[2] | 0.3 ng/L | 1.05 ng/L | Th[2] | 0.05 ng/L | 0.175 ng/L |
| Hf[2] | 2.5 ng/L | 8.75 ng/L | Ti[2] | 0.013 µg/L | 0.0455 µg/L |
| Ho[2] | 0.1 ng/L | 0.35 ng/L | Tm[2] | 0.1 ng/L | 0.35 ng/L |
| K[1] | 8.9 µg/L | 31.15 µg/L | U[2] | 0.03 ng/L | 0.105 ng/L |
| La[2] | 0.6 ng/L | 2.1 ng/L | V[2] | 0.038 µg/L | 0.133 µg/L |
| Li[1] | 0.6 µg/L | 2.1 µg/L | W[2] | 3.2 ng/L | 11.2 ng/L |
| Lu[2] | 0.03 ng/L | 0.105 ng/L | Y[2] | 1.1 ng/L | 3.85 ng/L |
| Mg[1] | 3.9 µg/L | 13.65 µg/L | Yb[2] | 0.02 ng/L | 0.07 ng/L |
| Mn[1] | 0.3 µg/L | 1.05 µg/L | Zn[1] | 4.7 µg/L | 16.45 µg/L |
| Mo[2] | 94.8 ng/L | 331.8 ng/L | Zr[2] | 4.9 ng/L | 17.15 ng/L |

[1] determined by ICP-OES.

[2] determined by ICP-MS.

Table S2. LOD and LOQ for the elements determined with ICP-OES and ICP-MS.
