# Supplementary figures and images for: On the Traceability of the Hazelnut Production Chain by Means of Trace Elements
Source: Molecules. 2022 Jun 15;27(12):3854. doi: 10.3390/molecules27123854 (PMC9228825; doi:10.3390/molecules27123854)

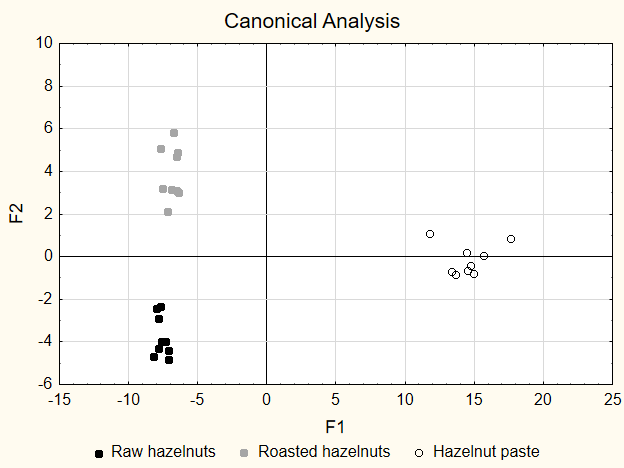

Supplement: Supplementary file 1 [file molecules-27-03854-s001.zip › Figure S1.tif]
